# Erratum

**Published:** 2022-04

In manuscript “Lack of association between fat mass and obesity-associated genetic variant (rs8050136) and type 2 diabetes mellitus” Saudi Med J 2022; Vol. 43 (2): 132-138 doi: 10.15537/smj.2022.43.2.20210822. The *p*-value result in Table 1, first row should appeared as: 0.2651.

In manuscript “Lack of association between fat mass and obesity-associated genetic variant (rs8050136) and type 2 diabetes mellitus” Saudi Med J 2022; Vol. 43 (2): 132-138 doi: 10.15537/smj.2022.43.2.20210822. The sentence in the result section page 134 line 3 and 4 should appeared as: The T2DM patient group was insignificantly older than the control group (*p*>0.005).

